# The association between ionized calcium level and 28-day mortality in patients with sepsis: a cohort study

**DOI:** 10.1038/s41598-025-05090-1

**Published:** 2025-07-02

**Authors:** Dan Niu, Huihui Bai, Yuan Zong

**Affiliations:** Department of Intensive Care Unit, Shaanxi Provincial People’s Hospital, 256 Youyi West Road, Xi’an, 710068 Shaanxi China

**Keywords:** Calcium, Sepsis, Mortality, Database, Diseases, Medical research, Risk factors

## Abstract

Ionized calcium is crucial for normal physiological function, and blood calcium disorders are associated with poor prognosis in various diseases. However, the relationship between ionized calcium and outcomes in sepsis patients remains understudied. This study aimed to investigate the association between ionized calcium levels and 28-day mortality in sepsis patients. This was a retrospective cohort study of patients with sepsis in the eICU Collaborative Research Database (eICU-CRD) from 208 distinct ICUs across the United States between 2014 and 2015 that explored, and ultimately we included 4,031 patients with sepsis. Ionized calcium level was the exposure variable, and 28-day mortality after ICU admission was the outcome variable. Binary logistic regression was used to explore the independent effects of ionized calcium on 28-day mortality using nonadjusted and multivariable-adjusted models. A generalized additive model and smoothed curve fitting were used to investigate the non-linear relationship between ionized calcium levels and 28-day mortality. A two-piecewise linear model was used to calculate the odds ratio (OR) and 95% confidence interval (CI) on either side of the inflection point. The remaining 4031 patients with sepsis were enrolled in the final analysis. The mean age of the patients was 64.30 ± 15.93 years, and 2167 patients (53.76%) were males. The overall incidence of 28-day death was 13.05% (526/4031). There was a nonlinear relationship between ionized calcium levels and 28-day mortality in patients with sepsis. When the ionized calcium levels were less than 4.25 mg/dL, the risk of 28-day mortality increased by 51% for every unit of decrease in ionized calcium (OR = 0.49, 95% CI 0.29–0.82, *P*-value = 0.0066). When the ionized calcium level was greater than 4.65 mg/dL, the risk of 28-day mortality increased by 69% for every unit of increase in ionized calcium (OR = 1.69, 95% CI 1.05–2.72, *P*-value = 0.0307). For patients with sepsis, the association between the ionized calcium levels and the 28-day mortality risk was a U-shaped curve. A higher or lower ionized calcium level was associated with an increased risk of 28-day mortality in patients with Sepsis.

**Supplementary Information:**

The online version contains supplementary material available at 10.1038/s41598-025-05090-1.

## Introduction

Sepsis is a global health problem, affecting hundreds of millions of people annually, with a mortality rate of around 30%^1,2^. It is a serious medical condition characterized by a dysregulated immune response to infection, leading to life-threatening organ dysfunction^3^. Even those who survive sepsis often face long-term challenges, including functional impairments and diminished quality of life^4^. So early recognition is crucial to successful treatment of sepsis with anti-infectives, intravenous fluids, and circulatory support^5^. In recent years, research on risk factors for poor prognosis in patients with sepsis has mainly focused on the following aspects: (1) underlying diseases and immune status, such as chronic liver disease^6^, malignancy and immunosuppression^7^; (2) infecting pathogens, with Gram-negative bacteria^8^ and fungi^6,8^ associated with worse outcomes; (3) clinical presentation and physiological parameters including age, blood urea nitrogen, albumin levels, high SOFA scores^9^, lactate levels^10^, and serum calcium levels^11^; (4) complications, such as acute respiratory distress syndrome^12^ and acute kidney injury^13^; and (5) medical-related factors, including ICU-acquired infections^14^, health care exposuresand^15^, delayed antimicrobial therapy^16^. Comprehensive assessment of these factors can help identify high-risk sepsis patients and improve their prognosis.

Derangements in ionized calcium levels occur very commonly in patients in the intensive care unit, especially those with sepsis^17^. Calcium plays a variety of essential physiological roles, including involvement in cell signaling, neurotransmission, muscle contraction (cardiac, smooth, and skeletal), and as a cofactor in enzymatic reactions such as blood coagulation^17^. Calcium in the blood primarily exists in three fractions—50% is protein-bound, 10% is in a diffusible but nonionized chelated form, and the remaining 40% is in the ionized form. The ionized calcium fraction is the most physiologically active and is the form that is regulated by the body and available for cellular utilization^18^. Measuring ionized calcium is preferred, especially in the intensive care unit (ICU) setting, as it is not affected by changes in albumin or acid–base status, which are common in critically ill patients^17,19^. Appropriate ionized calcium concentration levels are essential for the proper functioning of these physiological processes, and any abnormalities can lead to serious clinical consequences, such as cardiovascular complications and organ dysfunction^20,21^. Calcium can be a double-edged sword, playing a crucial role in maintaining normal physiological function while also potentially leading to cellular injury in certain circumstances. Calcium also regulates processes that can damage and kill cells, such as the activation of digestive enzymes, the release of cytokines, the generation of free radicals, the inhibition of ATP synthesis, and vasoconstriction^19^. Ghafouri E et al. reported that in some diseases, such as acute respiratory distress syndrome, cancer, COVID-19 and sepsis, hypocalcemia is a common symptom, and it may have both protective and detrimental effects^22^. However, the relationship between ionized calcium and outcomes in sepsis patients remains understudied. Yan D et al. reported lower or higher serum calcium levels were associated with increased risk of 28-day mortality in septic patients^11^. Lucas CE et al. found that severe sepsis may lead to multiple organ failure, which subsequently induces hyperparathyroidism and hypercalcemia, thereby posing a life-threatening risk of bradycardia^23^. The aim of this study was to investigate the relationship between ionized calcium levels and 28-day mortality in patients with sepsis.

## Materials and methods

### Data source

This was a retrospective observational study that utilized data extracted from the eICU Collaborative Research Database (eICU-CRD)^24^. The eICU-CRD is a multi-center ICU database containing highly granular data for over 200,859 admissions to ICUs monitored by eICU programme across the United States. During the period from 2014 to 2015, all data were automatically stored through the Philips Healthcare eICU program and retrieved electronically^24^.

Access to the eICU-CRD database is granted upon passing an examination and obtaining certification in accordance with the data usage agreement established by the PhysioNet Review Board. The database is released under the Health Insurance Portability and Accountability Act (HIPAA) Safe Harbor provision. Approval to access the data was obtained after completing the Collaborative Institutional Training Initiative (CITI) program “Data or Specimens Only Research”. The retrospective nature of this study, the lack of direct patient intervention, and the security schema certified by Privacert (Cambridge, MA) as meeting safe harbor standards, resulted in the study being exempt from institutional review board approval at the Massachusetts Institute of Technology (record ID: 58868345). For the same reasons, informed consent from patients was waived.

### Patient selection

**Fig. 1:**
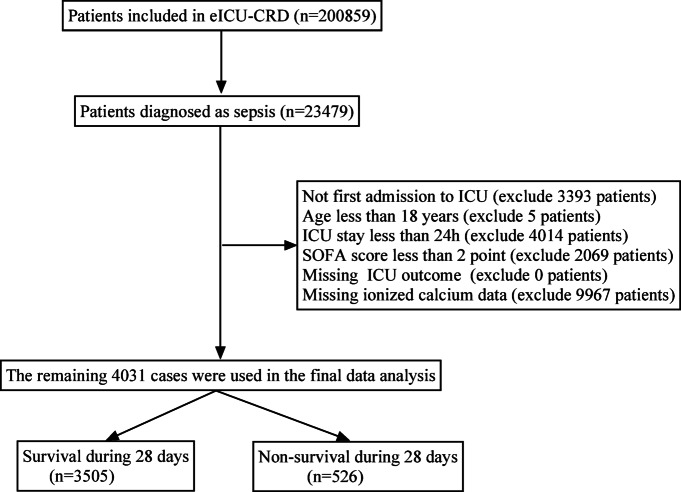
Flow chart of study population.

The medical records of 200,859 patients were found in the eICU-CRD. 23,479 patients diagnosed with sepsis on admission to the ICU were included. The diagnosis of sepsis was established according to the Third International Consensus Definitions for Sepsis and Septic Shock (Sepsis-3)^25^. Sepsis is defined as a Sequential Organ Failure Assessment (SOFA) score of ≥ 2, with the presence of infection or suspected infection. The diagnostic methodology was based on the diagnosis strings in accordance with the International Classification of Diseases 9th Revision (ICD-9) codes found in the diagnosis table of the eICU-CRD. The following exclusion criteria were used: (1) not first ICU admission, (2) ICU stay < 24 h, (3) < 18 years old, (4) SOFA score < 2 points, (5) missing ICU outcome, and (6) missing ionized calcium after ICU admission. The study flowchart was presented in Fig. 1.

### Variables and outcome measures

Structured Query Language (SQL) was used to extract the data; SQL cords were obtained from https://github.com/mit-lcp/eicu-code. All participant data within the first 24 h after ICU admission were collected from the eICU-CRD. The study collected physiological variables, baseline characteristics, blood gas analysis, laboratory indices, comorbidities, interventions, and severity scores from various tables in the database. The exposure variable was the ICU admission serum ionized calcium, defined as the first ionized calcium measured within 24 h of ICU admission.

Specifically: Physiological variables, including temperature, respiratory rate, heart rate, and mean arterial pressure (MAP), were obtained from the apacheApsVar table. Baseline characteristics, such as age, gender, ethnicity, height, and weight, were collected from the patient tables. Blood gas analysis, including pH, arterial partial pressure of oxygen (PaO_2_), partial pressure of arterial carbon dioxide (PaCO_2_), and lactate, were extracted from the laboratory table. Laboratory indices, including white blood cell count (WBC), hemoglobin, platelet, creatinine, blood urea nitrogen (BUN), sodium, potassium, ionized calcium, total bilirubin, albumin, glucose, and lactate, were collected from the laboratory table. Comorbidities, including chronic obstructive pulmonary disease (COPD), diabetes, hypertension, congestive heart failure (CHF), myocardial infarction (MI), chronic kidney disease (CKD), cirrhosis, cancer, pulmonary embolism (PE), and stroke, were extracted from the diagnosis table. Interventions, such as mechanical ventilation (MV), dialysis, vasopressor, and calcium gluconate or calcium chloride were collected from the treatment and infusiondrug tables. Severity at admission was measured by the sequential organ failure assessment (SOFA) score and APACHE IV score. The calculation method of SOFA score was shown in the Supplemental Table 1. APACHE IV score was collected from the apachePatientResult table. These two scores were estimated for all patients within 24 h of ICU admission. Septic shock was also extracted from the diagnostic table. If a variable was measured multiple times during the study, the first record was used for analysis. The outcome of the study was all-cause ICU mortality within 28 days after admission to the ICU.

**Table 1:** Baseline characteristics of patients according to ionized calcium.

	Total n = 4031	Ionized calcium (mg/dL)			*P*-value
		< 4.4 n = 1348	> = 4.4, < 5.2 n = 2351	> = 5.2 n = 332	
Demographics					
Age (year)	64.30 ± 15.93	61.92 ± 15.68	65.55 ± 16.03	64.77 ± 14.40	< 0.001
Gender					0.668
Male	2167 (53.76%)	733 (54.38%)	1346 (53.63%)	88 (50.87%)	
Female	1864 (46.24%)	615 (45.62%)	1164 (46.37%)	85 (49.13%)	
BMI (kg/m2)	29.27 ± 9.14	29.13 ± 8.77	29.35 ± 9.27	29.16 ± 10.11	0.777
Ethnicity					0.055
Caucasian	3149 (78.12%)	1042 (77.30%)	1977 (78.76%)	130 (75.14%)	
African American	488 (12.11%)	155 (11.50%)	304 (12.11%)	29 (16.76%)	
Hispanic	99 (2.46%)	33 (2.45%)	64 (2.55%)	2 (1.16%)	
Asian	89 (2.21%)	33 (2.45%)	56 (2.23%)	0 (0.00%)	
Native American	20 (0.50%)	10 (0.74%)	9 (0.36%)	1 (0.58%)	
Other/Unknown	186 (4.61%)	75 (5.56%)	100 (3.98%)	11 (6.36%)	
Site of infection					< 0.001
Pulmonary	1049(26.02%)	302(22.40%)	713(28.41%)	34(19.65%)	
Renal/UTI (including bladder)	552(13.69%)	168(12.46%)	362(14.42%)	22(12.72%)	
GI	375(9.30%)	165 (12.24%)	195(7.77%)	15(8.67%)	
Unknown	1602(39.74%)	561(41.62%)	962(38.33%)	79(45.66%)	
Cutaneous/soft tissue	242(6.00%)	77(5.71%)	156(6.22%)	9(5.20%)	
Other	204(5.06%)	71 (5.27%)	119 (4.74%)	14(8.09%)	
Gynercologic	7(0.17%)	4(0.30%)	3(0.12%)	0(0.00%)	
Vital signs					
Temperature (℃)	36.52 ± 1.42	36.45 ± 1.49	36.57 ± 1.39	36.30 ± 1.19	0.008
Heart rate (bpm)	100.55 ± 22.44	103.44 ± 22.63	99.22 ± 22.11	97.29 ± 23.49	< 0.001
Respiratory rate(bpm)	32 (18–39)	33(24–41)	31(15–38)	33(15–40)	< 0.001
MAP (mmHg)	56(47–120)	55(46–125)	56(47–117)	57(45.50–118)	0.515
Blood gas analysis					
pH	7.34 ± 0.11	7.32 ± 0.13	7.35 ± 0.11	7.34 ± 0.11	< 0.001
PaO_2_ (mmHg)	94 (72–140)	94(71–146)	93.50(72–136.60)	96.10(72–143.90)	0.883
PaCO_2_ (mmHg)	37(30.80–45.52)	36.00(29.05–44.00)	37.50(31.10–46)	39.25(31.62–48.30)	< 0.001
Lactate (mmol/L)	1.90(1.20–3.20)	2.30(1.30–4.10)	1.70(1.10–2.80)	1.60(1.10–2.60)	< 0.001
Laboratory parameters					
WBC (K/uL)	13.32(8.50–19.40)	13.16(8.10–20.38)	13.40(8.83–18.82)	14.10(8.24–19.30)	0.996
Hemoglobin (g/dL)	10.45 ± 2.32	10.56 ± 2.50	10.42 ± 2.22	10.11 ± 2.21	0.031
Platelets (K/uL)	178(118–254)	166(101–243)	184(126.25–258.75)	183(118–259)	< 0.001
Totail bilirubin (mg/dL)	0.70 (0.40–1.30)	0.80 (0.50–1.60)	0.60 (0.40–1.10)	0.60 (0.40–1.10)	< 0.001
Albumin (g/dL)	2.52 ± 0.67	2.42 ± 0.67	2.57 ± 0.66	2.60 ± 0.68	< 0.001
Glucose (mg/dL)	130(103–176)	132 (103–178.25)	129 (102–173)	132(106–177)	0.543
BUN (mg/dL)	30 (18–49)	32 (19.50–54)	28 (17–40)	34 (23–49)	< 0.001
Creatinine (mg/dL)	1.49 (0.91–2.67)	1.83 (1.08–3.38)	1.31 (0.88–2.27)	1.50 (0.95–2.82)	< 0.001
Sodium (mmol/L)	138.19 ± 6.50	137.21 ± 6.62	138.60 ± 6.30	139.81 ± 7.35	< 0.001
Potassium (mmol/L)	4.13 ± 0.83	4.16 ± 0.91	4.11 ± 0.79	4.18 ± 0.86	0.160
Chloride (mmol/L)	105.05 ± 7.74	103.93 ± 8.10	105.54 ± 7.44	106.66 ± 8.26	< 0.001
Ionized calcium (mg/dL)	4.47 ± 0.55	3.92 ± 0.35	4.68 ± 0.24	5.81 ± 0.70	< 0.001
Severity of illness					
APACHE IV score	77.65 ± 27.99	84.00 ± 30.30	74.19 ± 26.13	78.33 ± 26.74	< 0.001
SOFA	7.03 ± 3.31	7.91 ± 3.57	6.55 ± 3.06	7.10 ± 3.31	< 0.001
Septic shock	608 (15.08%)	273(20.25%)	306 (12.19%)	29 (16.76%)	< 0.001
Interventions					
Mechanical ventilation	1832 (45.45%)	721 (53.49%)	1036 (41.27%)	75 (43.35%)	< 0.001
Dialysis	518 (12.85%)	254 (18.84%)	240 (9.56%)	24 (13.87%)	< 0.001
Vasopressor	532 (13.20%)	210 (15.58%)	294 (11.71%)	28 (16.18%)	0.002
Calcium gluconate/calcium chloride	225(5.58%)	126 (9.35%)	91 (3.63%)	8 (4.62%)	< 0.001
Comorbidity					
COPD	822 (20.39%)	218 (16.17%)	571 (22.75%)	33 (19.08%)	< 0.001
Diabetes	995 (24.68%)	296 (21.96%)	650 (25.90%)	49 (28.32%)	0.014
Hypertension	2382 (59.09%)	768 (56.97%)	1519 (60.52%)	95 (54.91%)	0.053
CHF	899 (22.30%)	263 (19.51%)	594 (23.67%)	42 (24.28%)	0.010
MI	450 (11.16%)	158 (11.72%)	277 (11.04%)	15 (8.67%)	0.461
CKD	396 (9.82%)	138 (10.24%)	240 (9.56%)	18 (10.40%)	0.771
Cirrhosis	208 (5.16%)	94 (6.97%)	103 (4.10%)	11 (6.36%)	< 0.001
Cancer	797 (19.77%)	269 (19.96%)	487 (19.40%)	41 (23.70%)	0.382
PE	148 (3.67%)	61 (4.53%)	81 (3.23%)	6 (3.47%)	0.122
Stroke	511 (12.68%)	161 (11.94%)	332 (13.23%)	18 (10.40%)	0.342
28-Day mortality					< 0.001
No	3505 (86.95%)	1104 (81.90%)	2256 (89.88%)	145 (83.82%)	
Yes	526 (13.05%)	244 (18.10%)	254 (10.12%)	28 (16.18%)	

Data are expressed as Mean ± SD, Median (Q1–Q3) or N (%). Among the 4031 patients, the amount of missing values for the covariates were 107 (2.65%) for BMI, 209 (5.18%) for temperature, 47 (1.17%) for heart rate, 51 (1.27%) for respiratory rate, 37 (0.92%) for MAP, 678 (16.82%) for pH, 695 (17.24%) for PaO_2_, 707 (17.54%) for PaCO_2_, 515 (12.78%) for lactate, 25 (0.62%) for WBC, 10 (0.25%)for hemoglobin, 43 (1.07%)for platelets, 447 (11.09%) for total bilirubin, 266 (6.60%) for albumin, 7 (0.17%) for glucose, 8 (0.20%) for BUN, 14 (0.35%) for creatinine, 81 (2.01%) for sodium, 7 (0.17%) for potassium, 7 (0.17%) for chloride, 436 (10.82%) for APACHE IV score. BMI, body mass index; SOFA, the sequential organ failure assessment; UTI, urinary tract infection; GI, gastrointestinal infection; MAP, mean arterial pressure; PaO_2_, arterial partial pressure of oxygen, PaCO_2_, partial pressure of arterial carbon dioxide); WBC, white blood cell count; BUN, blood urea nitrogen; MV, mechanical ventilation; COPD, chronic obstructive pulmonary disease; CHF, congestive heart failure; MI, myocardial infarction; CKD, chronic kidney disease; PE, pulmonary embolism.

### Statistical analysis

Data on categorical variables are presented as percentages, and data on continuous variables are expressed as mean (SD) or interquartile range (IQR). As this was a cohort study, the exposure variables were divided into three groups according to the clinical normal range, and the baseline characteristics of the patients were distributed differently among the groups. One-way analysis of variance (ANOVA) (normal distribution), the Kruskal–Wallis test (non-normal distribution), and the chi-square test (categorical variables) were performed to evaluate statistically significant differences between the groups. Univariate and multivariable binary logistic regression models were also used to examine the relationship between ionized calcium levels and 28-day mortality in patients with sepsis.

Unadjusted models (no covariates adjusted, Model 1), minimally adjusted models (adjusted for gender, age, and BMI, Model 2), and fully adjusted models (adjust for gender, age, BMI, ethnicity, temperature, respiratory rate, heart rate, MAP, PaO_2_, PaCO_2_, WBC, hemoglobin, total bilirubin, albumin, glucose, BUN, creatinine, sodium, potassium, chloride, dialysis, vasopressor, calcium gluconate/calcium chloride, septic shock, COPD, diabetes, hypertension, CHF, MI, CKD, cirrhosis, cancer, PE, stroke, site of infection, APACHE IV score and SOFA score, Model 3) were presented at a 95% confidence interval, and the odds ratio (OR) was presented at a 95% confidence interval. The ionized calcium levels were first classified to increase the reliability of the study results. A trend test was then performed to ascertain whether the data were consistent with the continuous or categorical variables. Covariates were included as potential confounding variables in the final models if they changed the effects of ionized calcium level on 28-day mortality by more than 10% or were significantly associated with 28-day mortality. Because ionized calcium level is a continuous variable, it is impossible to exclude a nonlinear relationship. Considering the limitations of binary logistic regression models in handling nonlinear relationships, a generalized additive model (GAM) was employed to investigate the relationship between ionized calcium levels and 28-day mortality in patients with sepsis. If there was a nonlinear relationship, the inflection point value was calculated using a recursive algorithm, and a two-piecewise linear model was used to calculate the OR at a 95% confidence interval on either side of the inflection point. Furthermore, subgroup analysis was performed to determine whether there were differences between subgroups in the prediction of clinical outcomes based on ionized calcium levels. Interactions between subgroups were examined using the log-likelihood ratio test. We used multiple imputation, based on 5 replications and a chained equation approach method in the R MI procedure, to account for missing data. Statistical significance was defined as a two-tailed P-value of smaller than 0.05. All data processing steps and statistical tests were performed using EmpowerStats (www.empowerstats.com; X&Y Solutions Inc.) and statistical software package R (The R Foundation; http://www.r-project.org; version 3.4.3).

## Results

### Selection of participants

Among the 200,859 patients identified from eICU-CRD, 23,479 patients were diagnosed with sepsis. We excluded 3393 patients with duplicate ICU admission, 4,014 patients with ICU stays < 24 h, 5 patients aged < 18 years, 2069 patients with SOFA scores < 2 point, and 9967 patients with missing ionized calcium data. The remaining 4,031 patients with sepsis were enrolled in the final analysis. The overall incidence of 28-day death was 13.05% (526/4031) (Fig. 1).

### Baseline characteristics of patients

The baseline characteristics of 4,031 patients were enrolled and categorized based on their ionized calcium levels into three groups: < 4.4 mg/dL (n = 1348), ≥ 4.4 mg/dL and < 5.2 mg/dL (n = 2351), and ≥ 5.2 mg/dL (n = 332) in Table 1. The table compared the patient’s demographics, site of infection, vital signs, laboratory parameters, severity of illness, interventions, comorbidities, and 28-day mortality across different levels of ionized calcium. The average age of the overall cohort was 64.30 ± 15.93 years, with the < 4.4 mg/dL group being significantly younger (61.92 ± 15.68 years) compared to the ≥ 4.4 mg/dL and < 5.2 mg/dL (65.55 ± 16.03 years) and ≥ 5.2 mg/dL (64.77 ± 14.40 years) groups (*P* < 0.001). Males comprised 53.76% of the population (n = 2167), with no significant gender differences between groups (*P* = 0.668). The mean BMI was 29.27 ± 9.14 kg/m^2^, showing no significant difference across groups (*P* = 0.777). Ethnic distribution showed a prevalence of Caucasians at 78.12% (n = 3149), with borderline significance noted across groups (*P* = 0.055). Infection site distribution shows significant variation, especially in pulmonary infections, where 28.41% of patients in the intermediate group are affected, compared to the other two groups (*P* < 0.001). Vital signs revealed significant differences in heart rate (*P* < 0.001) and respiratory rate (*P* < 0.001) among the groups. Moreover, biochemical parameters such as pH (*P* < 0.001) and lactate levels (*P* < 0.001) also displayed significant variations. Additionally, APACHE IV and SOFA scores are significantly higher in the low calcium group (84.00 ± 30.30 and 7.91 ± 3.57) compared to the others, indicating greater severity of illness (*P* < 0.001). Patients requiring interventions such as mechanical ventilation (53.49% in low calcium vs. 41.27% and 43.35% in higher groups, *P* < 0.001) also show higher rates of dialysis (18.84% vs. 9.56% and 13.87%, *P* < 0.001). COPD was more prevalent in the the the intermediate group (22.75%, *P* < 0.001) compared to other groups, while having lower rates of cirrhosis (4.10%, *P* < 0.001). Notably, the 28-day mortality rate is significantly lower in the intermediate group (10.12% vs. 18.10% and 16.18%, *P* < 0.001).

### Univariate analysis of risk factors associated with 28-day mortality in patients with sepsis

The univariate logistic regression analysis of 28-day mortality in all patients is presented in Table 2. The results show that older age (OR 1.01, 95% CI 1.01–1.02, *P*-value < 0.0001), lower BMI (OR 0.98, 95% CI 0.97–0.99, *P*-value = 0.0018), higher illness severity scores (SOFA score: OR 1.23, 95% CI 1.19–1.26, *P*-value < 0.0001; APACHE IV score: OR 1.03, 95% CI 1.03–1.04, *P*-value < 0.0001), and abnormal physiological parameters (lower temperature: OR 0.84, 95% CI 0.79–0.90, *P*-value < 0.0001; higher heart rate: OR 1.01, 95% CI 1.00–1.01, *P* = 0.0156; higher respiratory rate: OR 1.01, 95% CI 1.00–1.01, *P*-value = 0.0265) were significant predictors of increased mortality. Various laboratory derangements, including higher lactate (OR 1.22, 95% CI 1.18–1.26, *P*-value < 0.0001), WBC (OR 1.01, 95% CI 1.00–1.02, *P*-value = 0.0017), and bilirubin (OR 1.10, 95% CI 1.07–1.13, *P*-value < 0.0001), as well as lower platelets (OR 1.00, 95% CI 1.00–1.00, *P*-value = 0.0001) and albumin (OR 0.61, 95% CI 0.53–0.71, *P*-value < 0.0001), were also independently associated with higher 28-day mortality. Additionally, the use of mechanical ventilation (OR 3.53, 95% CI 2.89–4.31, *P*-value < 0.0001), dialysis (OR 2.40, 95% CI 1.91–3.02, *P*-value < 0.0001), and vasopressors (OR 1.64, 95% CI 1.29–2.09, *P*-value < 0.0001), as well as the presence of septic shock (OR 1.57, 95% CI 1.25–1.98, *P*-value = 0.0001) and certain comorbidities like chronic kidney disease (OR 1.44, 95% CI 1.09–1.90, *P*-value = 0.0107) and cirrhosis (OR 2.23, 95% CI 1.60–3.10, *P*-value < 0.0001), were also independently associated with higher 28-day mortality.

**Table 2:** Univariate logistics regression analysis of all patients on 28-day mortality.

Variables	28-Day mortality OR 95% CI *P*-value
Gender	
Male	Reference
Female	0.99 (0.82, 1.19) 0.9081
Age (year)	1.01 (1.01, 1.02) < 0.0001
BMI (kg/m2)	0.98 (0.97, 0.99) 0.0018
Ethnicity	
Caucasian	Reference
African American	1.01 (0.76, 1.34) 0.9450
Hispanic	0.94 (0.51, 1.73) 0.8427
Asian	1.17 (0.64, 2.12) 0.6153
Native American	1.20 (0.35, 4.12) 0.7693
Other/Unknown	1.42 (0.95, 2.10) 0.0843
Site of infection recoded	
Pulmonary	Reference
Renal/UTI (including bladder)	0.64 (0.46, 0.90) 0.0109
GI	1.16 (0.83, 1.61) 0.3815
Unknown	1.04 (0.83, 1.30) 0.7637
Cutaneous/soft tissue	0.57 (0.35, 0.93) 0.0250
Other	1.01 (0.65, 1.56) 0.9716
Gynercologic	1.06 (0.13, 8.84) 0.9599
SOFA score	1.23 (1.19, 1.26) < 0.0001
APACHE IV score	1.03 (1.03, 1.04) < 0.0001
Temperature (℃)	0.84 (0.79, 0.90) < 0.0001
Heart rate (bpm)	1.01 (1.00, 1.01) 0.0156
Respiratory rate (bpm)	1.01 (1.00, 1.01) 0.0265
MAP (mmHg)	1.00 (1.00, 1.00) 0.8074
pH	0.04 (0.02, 0.10) < 0.0001
PaO_2_ (mmHg)	1.00 (1.00, 1.00) 0.0055
PaCO_2_ (mmHg)	1.00 (0.99, 1.00) 0.3403
Lactate (mmol/L)	1.22 (1.18, 1.26) < 0.0001
WBC (K/uL)	1.01 (1.00, 1.02) 0.0017
Hemoglobin (g/dL)	0.98 (0.94, 1.02) 0.2381
Platelets(K/uL)	1.00 (1.00, 1.00) 0.0001
Total bilirubin (mg/dL)	1.10 (1.07, 1.13) < 0.0001
Albumin (g/dL)	0.61 (0.53, 0.71) < 0.0001
Glucose (mg/dL)	1.00 (1.00, 1.00) 0.8369
BUN (mg/dL)	1.01 (1.00, 1.01) 0.0003
Creatinine (mg/dL)	1.06 (1.01, 1.10) 0.0150
Sodium (mmol/L)	1.00 (0.98, 1.01) 0.6739
Potassium (mmol/L)	1.10 (0.99, 1.23) 0.0818
Chloride (mmol/L)	1.00 (0.99, 1.01) 0.9479
Ionized calcium (mg/dL)	0.67 (0.56, 0.79) < 0.0001
MV	
No	Reference
Yes	3.53 (2.89, 4.31) < 0.0001
Dialysis	
No	Reference
Yes	2.40 (1.91, 3.02) < 0.0001
Vasopressor	
No	Reference
Yes	1.64 (1.29, 2.09) < 0.0001
Calcium gluconate/calcium chloride	
No	Reference
Yes	1.29 (0.89, 1.86) 0.1773
Septic shock	
No	Reference
Yes	1.57 (1.25, 1.98) 0.0001
COPD	
No	Reference
Yes	1.09 (0.87, 1.37) 0.4343
Diabetes	
No	Reference
Yes	0.83 (0.67, 1.04) 0.1080
Hypertension	
No	Reference
Yes	0.87 (0.72, 1.04) 0.1326
CHF	
No	Reference
Yes	0.93 (0.75, 1.17) 0.5510
MI	
No	Reference
Yes	0.88 (0.65, 1.19) 0.3960
CKD	
No	Reference
Yes	1.44 (1.09, 1.90) 0.0107
Cirrhosis	
No	Reference
Yes	2.23 (1.60, 3.10) < 0.0001
Cancer	
No	Reference
Yes	1.35 (1.08, 1.67) 0.0071
PE	
No	Reference
Yes	1.30 (0.83, 2.04) 0.2451
Stroke	
No	Reference
Yes	1.37 (1.06, 1.77) 0.0153

### Relationship between ionized calcium and 28-day mortality in patients with sepsis

Different covariate adjustment strategies were used to elucidate the relationship between ionized calcium levels and 28-day mortality in patients with sepsis. The nonadjusted and adjusted models are presented in Table 3. The non-adjusted analysis showed that higher ionized calcium levels were associated with lower odds of mortality (OR 0.67, 95% CI 0.56–0.79, *P*-value < 0.0001). This relationship persisted after adjusting for potential confounders in Model I (OR 0.63, 95% CI 0.53–0.76, *P*-value < 0.0001) and Model II (OR 0.78, 95% CI 0.62–0.98, P-value = 0.0319). Sensitivity analysis was performed using categorical variables and *P*-values were calculated for the trend tests. However, inconsistent results were obtained when comparing ionized calcium levels as continuous or categorical variables (Table 3). The unequal variances in OR values between the different ionized calcium groups suggested a nonlinear relationship between ionized calcium levels and 28-day mortality.

**Table 3:** Relationship between ionized calcium and 28-day mortality in patients with sepsis.

Exposure	Non-adjusted OR 95%CI *P*-value	Adjust I OR 95%CI *P*-value	Adjust II OR 95%CI *P*-value
Ionized calcium	0.67 (0.56, 0.79) < 0.0001	0.63 (0.53, 0.76) < 0.0001	0.78 (0.62, 0.98) 0.0319
Ionized calcium group			
< 4.4	1.96 (1.62, 2.37) < 0.0001	2.11 (1.74, 2.56) < 0.0001	1.68 (1.32, 2.15) < 0.0001
> = 4.4, < 5.2	Reference	Reference	Reference
> = 5.2	1.72 (1.12, 2.62) 0.0128	1.82 (1.18, 2.79) 0.0063	1.92 (1.15, 3.21) 0.0122
*P* for trend	< 0.0001	< 0.0001	< 0.0001

Non-adjusted model adjust for: None.

Adjust I model adjust for: gender, age, BMI.

Adjust II model adjust for: gender, age, BMI, ethnicity, temperature, respiratory rate, heart rate, MAP, PaO_2_, PaCO_2_, WBC, hemoglobin, total bilirubin, albumin, glucose, BUN, creatinine, sodium, potassium, chloride, dialysis, vasopressor, calcium gluconate/calcium chloride, septic shock, COPD, diabetes, hypertension, CHF, MI, CKD, cirrhosis, cancer, PE, stroke, site of infection, APACHE IV score and SOFA score.

**Fig. 2:**
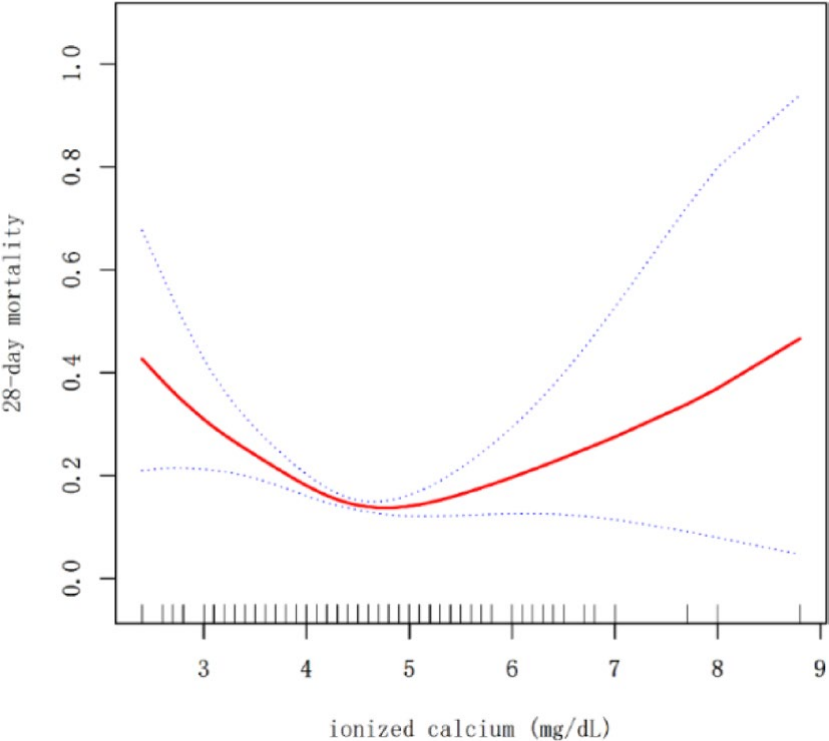
Association between ionized calcium and 28-day mortality. There was a U-shaped relationship between ionized calcium and 28-day mortality in patients with sepsis (Adjusted for: gender, age, BMI, ethnicity, temperature, respiratory rate, heart rate, MAP, PaO_2_, PaCO_2_, WBC, hemoglobin, total bilirubin, albumin, glucose, BUN, creatinine, sodium, potassium, chloride, dialysis, vasopressor, calcium gluconate/calcium chloride, septic shock, COPD, diabetes, hypertension, CHF, MI, CKD, cirrhosis, cancer, PE, stroke, site of infection, APACHE IV score and SOFA score.)

A generalized additive model was employed to explore the nonlinear association between ionized calcium levels and 28-day mortality. The findings revealed a U-shaped relationship between ionized calcium and 28-day mortality in patients with sepsis, after adjusting for the following covariates: gender, age, BMI, ethnicity, temperature, respiratory rate, heart rate, MAP, PaO_2_, PaCO_2_, WBC, hemoglobin, total bilirubin, albumin, glucose, BUN, creatinine, sodium, potassium, chloride, dialysis, vasopressor, calcium gluconate/calcium chloride, septic shock, COPD, diabetes, hypertension, CHF, MI, CKD, cirrhosis, cancer, PE, stroke, site of infection, APACHE IV score and SOFA score. (Fig. 2).

A significant nonlinear association existed between ionized calcium and 28-day mortality (P for log-likelihood ratiotest <0.001) (Table 4). Inflection points were determined using a two-stage linear model and recursive techniques. The results showed that the inflection points were 4.25 mg/dL and 4.65 mg/dL. The analyses revealed that the risk of 28-day mortality increased by 51% for every unit decrease in ionized calcium when levels were less than 4.25 mg/dL (OR=0.49, 95% CI 0.29-0.82, *P*-value=0.0066). Conversely, when ionized calcium was greater than 4.65 mg/dL, the risk of 28-day mortality increased by 69% for every unit increase (OR=1.69, 95% CI 1.05-2.72, *P*-value=0.0307) . No significant association was observed in the intermediate range (4.25–4.65 mg/dL: OR = 0.35, 95% CI: 0.08–1.59, *P* = 0.173) (Table 4).

**Table 4:** The addressing of nonlinear association between ionized calcium and 28-day mortality.

Outcome	OR 95%*CI*	*P*-value
Model I		
One line effect	0.78 (0.62, 0.98)	0.0319
Model II		
< 4.25	0.49 (0.29, 0.82)	0.0066
4.25–4.65	0.35 (0.08, 1.59)	0.1732
> 4.65	1.69 (1.05, 2.72)	0.0307
* P* value for LRT test*		< 0.001

Data were presented as OR (95% CI) P value; Model I, linear analysis; Model II, non-linear analysis. Adjusted for: gender, age, BMI, ethnicity, temperature, respiratory rate, heart rate, MAP, PaO_2_, PaCO_2_, WBC, hemoglobin, total bilirubin, albumin, glucose, BUN, creatinine, sodium, potassium, chloride, dialysis, vasopressor, calcium gluconate/calcium chloride, septic shock, COPD, diabetes, hypertension, CHF, MI, CKD, cirrhosis, cancer, PE, stroke, site of infection, APACHE IV score and SOFA score. *CI* confidence interval, OR odds ratio, LRT logarithm likelihood ratio test. *P < 0.05 indicates that model II is significantly different from Model I.

#### Subgroup analyses

The subgroup analyses for the relationship between ionized calcium and 28-day mortality in patients with sepsis were presented in Table 5, which were performed according to cirrhosis and septic shock. The results showed that in different subgroups, the relationship between ionized calcium and 28-day mortality in patients with sepsis stably existed after careful adjustments.

**Table 5:** Subgroup analysis of the associations between ionized calcium and 28-day mortality in patients with sepsis.

	N	ionized calcium (mg/dL)			*P* for interaction
		< 4.4 (OR, 95%CI, *P*-value)	> = 4.4, < 5.2 (OR, 95%CI, *P*-value)	> = 5.2 (OR, 95%CI, *P*-value)	
Cirrhosis					0.5427
No	3823	1.88 (1.54, 2.29) < 0.0001	1	1.58 (1.01, 2.49) 0.0466	
Yes	208	2.55 (1.28, 5.07) 0.0076	1	3.11 (0.81, 11.86) 0.0971	
Septic shock					0.0855
No	3423	2.10 (1.69, 2.59) < 0.0001	1	1.56 (0.95, 2.56) 0.0756	
Yes	608	1.29 (0.84, 1.99) 0.2388	1	2.05 (0.86, 4.89) 0.1067	

## Discussion

This retrospective cohort study found a higher or lower ionized calcium level was associated with a higher risk of 28-day mortality in patients with sepsis in the eICU-CRD database from 208 distinct ICUs across the United States between 2014 and 2015. The major finding was that the association between the ionized calcium level and the risk of 28-day mortality was U-shaped, and the risk was highest in those with very low or very high ionized calcium level. When the ionized calcium levels were less than 4.25 mg/dL, the risk of 28-day mortality increased by 51% for every unit of decrease in ionized calcium (OR = 0.49, 95% CI 0.29–0.82, *P*-value = 0.0066). When the ionized calcium level was greater than 4.65 mg/dL, the risk of 28-day mortality increased by 69% for every unit of increase in ionized calcium (OR = 1.69, 95% CI 1.05–2.72, *P*-value = 0.0307).

The relationship between serum calcium levels and mortality risk has been extensively studied. In the general population, both low and high serum calcium concentrations have been linked to increased all-cause mortality^26^. In the intensive care unit (ICU) setting, a non-linear U-shaped relationship was found between albumin-corrected calcium and 30-day in-hospital mortality^27^. Similarly, in patients with acute myocardial infarction, both decreased and increased corrected serum calcium were associated with increased in-hospital mortality^28^. Hypocalcemia has also been observed in other clinical conditions, such as pulmonary embolism, where it was an independent predictor of 30-day mortality^29^. In critically ill patients with cardiogenic shock, lower serum ionized calcium levels on admission were potential predictors of increased mortality risk^30^. Furthermore, lower serum calcium levels are associated with increased mortality risk in patients with severe acute osteomyelitis^31^. Lower ionized calcium was also linked to a higher likelihood of developing severe infection or sepsis^31^. In patients with hypertensive intracerebral hemorrhage, lower ionized calcium was associated with early hematoma expansion and poor outcome^32^. In very low birth weight infants with sepsis, ionized calcium levels correlated with the severity of sepsis and could serve as an independent predictor of poor prognosis^33^. Hypocalcemia was also common in neonates with sepsis and was significantly associated with organ dysfunction and sepsis-related mortality^34^. In hospitalized COVID-19 patients, hypocalcemia was frequent and could identify those with a worse prognosis^35^. In contrast, higher serum calcium levels at baseline were associated with increased 1-year all-cause mortality after ischemic stroke^36^. These findings, whether using ionized calcium, albumin-corrected total serum calcium or uncorrected serum calcium, consistently demonstrate a non-linear, U-shaped relationship between calcium levels and mortality risk across various patient populations, including the general population, intensive care unit patients, and acute myocardial infarction populations.

The findings of the present study are consistent with the results reported by Yan et al.^11^, which also utilized a retrospective cohort design to investigate the relationship between serum calcium levels and 28-day mortality in patients with sepsis. Both studies identified a U-shaped association, where both low and high calcium levels were associated with increased risk of mortality. Despite the similarities in study design and primary outcomes, there were some notable differences between the two investigations. The current study utilized the eICU-CRD database, which includes data from 208 distinct ICUs, while Yan et al. used the MIMIC-III database, a large, single-center, publicly available critical care database. This difference in data sources may have led to variations in patient populations and sample sizes between the two studies, potentially affecting the generalizability of the findings. An important distinction is that the current study utilized ionized calcium measurements, which reflect the physiologically active form and are not influenced by albumin levels or acid–base status. In contrast, Yan et al. used uncorrected serum calcium. Overall, the two studies are highly consistent in their patient population and outcome measures, further validating the relationship between low and high calcium levels and adverse outcomes in sepsis. The differences in data sources and exposure factors, however, highlight the need for additional multi-center studies to fully elucidate the complex interplay between calcium level and clinical outcomes in this critically ill patient population.

The mechanisms underlying the dysregulation in calcium dysregulation during sepsis may be multifaceted: Firstly, the inflammatory response triggered by sepsis can impact the secretion and activity of calcium-regulating hormones. Studies have shown that inflammatory cytokines, such as IL-1, IL-6, and TNF-α, can suppress the secretion of parathyroid hormone (PTH), leading to a decrease in blood calcium levels^37^. Additionally, the activation of vitamin D is also inhibited by inflammation, further exacerbating the imbalance in calcium metabolism^19^. Secondly, the oxidative stress and cellular damage induced by sepsis can lead to a disruption in the intracellular calcium homeostasis, with impaired calcium efflux from the cells. The resulting intracellular calcium overload can trigger cell apoptosis and organ dysfunction^38^. Furthermore, sepsis often leads to acute kidney injury, which can reduce glomerular filtration and alter tubular function^39^. Renal calcium excretion is regulated by two main mechanisms: tubular calcium reabsorption and filtered calcium load, and disruption of either or both of these mechanisms leads to abnormal calcium homeostasis^40^. The impaired ability of the kidneys to activate vitamin D also reduces their capacity to regulate calcium levels^41^. Finally, hypocalcemia after sepsis stimulates the release of PTH to restore normal calcium levels, but persistent sepsis combined with multiple organ failure can lead to sustained parathyroid hormone release, resulting in hypercalcemia and even life-threatening complications^42,43^. Understanding these potential mechanisms is crucial for emphasizing the monitoring of ionized calcium levels and the prognosis of patients with sepsis.

The study investigates the association between ionized calcium levels and 28-day mortality in sepsis patients, addressing a knowledge gap in this area. Previous research has indicated a correlation between calcium disorders and adverse outcomes in various diseases, yet data pertaining to sepsis patients remains sparse. Conducting a retrospective cohort analysis using data from 4,031 sepsis patients across 208 intensive care units, the study demonstrates a U-shaped relationship between ionized calcium levels and mortality. These findings not only validate earlier studies but also enhance the understanding of the physiological impact of calcium on sepsis outcomes, underscoring the necessity for monitoring this parameter in clinical practice. The research contributes a critical perspective to the field, offering insights for future studies aimed at exploring the underlying mechanisms linking ionized calcium levels and sepsis mortality.

This study demonstrates several strengths that enhance its contribution to understanding the relationship between ionized calcium levels and 28-day mortality in sepsis patients. The robust study design, featuring a large retrospective cohort of 4,031 patients sourced from the reliable eICU-CRD, ensures substantial statistical power and credibility. The clear inclusion and exclusion criteria further support the validity of the study population. Comprehensive data analysis was conducted using a variety of statistical methods, including binary logistic regression, generalized additive models, and piecewise linear models, allowing for a thorough exploration of the nonlinear relationship between ionized calcium levels and mortality. The study’s adjustments for potential confounding factors and the use of multiple imputation techniques to address missing data enhance the reliability of the findings. The identification of a U-shaped relationship between ionized calcium levels and 28-day mortality, provides valuable clinical insights. Additionally, the discussion of potential physiological mechanisms offers a foundation for future research. Overall, the study’s design, analysis, and interpretation collectively contribute to its significance in the field.

Our study had several limitations. First, the study population was derived from the eICU-CRD, which only includes ICU patients from the United States, potentially limiting the generalizability of the findings to other geographic regions and healthcare settings. Second, the study cohort was predominantly Caucasian, with relatively small proportions of other racial/ethnic groups, which may preclude a comprehensive assessment of potential disparities across diverse populations. Additionally, as an observational, retrospective cohort study, our analysis could only evaluate the association between ionized calcium levels and 28-day mortality. This design does not allow for establishing causal relationships or providing clinical treatment recommendations. While we adjust for confounding factors whenever possible, the potential influence of unmeasured or unknown confounders cannot be entirely excluded. Furthermore, certain clinically relevant variables, such as nutritional status, the stage of the cirrhosis, were not fully captured, which may have affected the accuracy of the results. Future prospective studies or randomized controlled trials with broader and more representative patient populations, as well as a more comprehensive assessment of potential mediating factors, would help to further elucidate the complex relationship between ionized calcium abnormalities and clinical outcomes in sepsis.

## Conclusion

For patients with sepsis, the association between the ionized calcium levels and the 28-day mortality risk was a U-shaped curve. A higher or lower ionized calcium level was associated with an increased risk of 28-day mortality in patients with sepsis.

## Electronic supplementary material

Below is the link to the electronic supplementary material.

Supplementary Material 1

## Data Availability

Data were fully available at https://eicu-crd.mit.edu/. The links is the direct persistent links to the datasets and researchers need to completed the Collaborative Institutional Training Initiative (CITI) program “Data or Specimens Only Research”.and obtained the certifcation prior to accession.
