# Supplementary material for: The association between ionized calcium level and 28-day mortality in patients with sepsis: a cohort study
Source: Sci Rep. 2025 Jul 2;15:22761. doi: 10.1038/s41598-025-05090-1 (PMC12216518; doi:10.1038/s41598-025-05090-1)
Supplement: Supplementary file 1 — Supplementary Material 1 [file 41598_2025_5090_MOESM1_ESM.docx]

**The association between** **Ionized Calcium Level and 28-day Mortality in Patients with Sepsis: A Cohort Study**

Dan Niu^1^, Huihui Bai^1^, Yuan Zong^1^*

^*^Corresponding author:

Yuan Zong

149309397@qq.com

^1^Department of Intensive Care Unit, Shaanxi Provincial People's Hospital, 256 Youyi West Road, Xi'an 710068, Shaani, China.

**Supplementary material**

Supplemental Table 1. Sequential Organ Failure Assessment (SOFA) Score

| System | variables | Score | | | | |
| --- | --- | --- | --- | --- | --- | --- |
|  |  | 0 | 1 | 2 | 3 | 4 |
| **Respiration** | PaO_2_/FiO_2_, mmHg | ≥400 | <400 (53.3) | <300 | <200  respiratory support | <100  respiratory support |
| **Coagulation** | Platelets, ×10^6^/mL | ≥150 | <150 | <100 | <50 | <20 |
| **Liver** | Bilirubin,mg/dL (μmol/L) | <1.2 (20) | 1.2–1.9 (20–32) | 2.0–5.9  (33–101) | 6.0–11.9  (102–204) | >12.0  (204) |
| **Cardiovascular** | MAP, mmHg | ≥70 | <70 |  |  |  |
|  | Dopamine^*^ |  |  | <5 | 5.1–15 | >15 |
|  | Dobutamine^*^ |  |  | any dose |  |  |
|  | Epinephrine^*^ |  |  |  | ≤0.1 | >0.1 |
|  | Norepinephrine^*^ |  |  |  | ≤0.1 | >0.1 |
| **Central nervous system** | Glasgow Coma Scale score^**^ | 15 | 13–14 | 10–12 | 6–9 | <6 |
| **Renal** | Creatinine, mg/dL | <1.2 | 1.2–1.9 | 2.0–3.4 | 3.5–4.9 | ≥5.0 |
|  | Urine output, mL/day |  |  |  | <500 | <200 |

^*^Catecholamine doses are given as μg/kg/min for at least 1 hour.

^**^ Glasgow Coma Scale scores range from 3-15; higher score indicates better neurological function.

Abbreviations: FiO_2_, fraction of inspired oxygen; MAP, mean arterial pressure; PaO_2_, arterial partial pressure of oxygen.

With missing data: The amount of missing values for the covariates were 107 (2.65%) for BMI, 209 (5.18%) for temperature, 47 (1.17%) for heart rate, 51 (1.27%) for respiratory rate, 37 (0.92%) for MAP, 678 (16.82%) for pH, 695 (17.24%) for PaO_2_, 707 (17.54%) for PaCO_2_, 515 (12.78%) for lactate, 25 (0.62%) for WBC, 10 (0.25%) for hemoglobin, 43 (1.07%) for platelets, 447 (11.09%) for total bilirubin, 266 (6.60%) for albumin, 7 (0.17%) for glucose, 8 (0.20%) for BUN, 14 (0.35%) for creatinine, 81 (2.01%) for sodium, 7 (0.17%) for potassium, 7 (0.17%) for chloride, 436 (10.82%) for APACHE Ⅳ score.

Dealing with missing data: We used multiple imputation, based on 5 replications and a chained equation approach method in the R MI procedure, to account for missing data.

Supplemental Table 2. Relationship between ionized calcium and 28-day mortality in patients with sepsis

| Exposure | With missing data  (n=2358) | | | Dealing with missing data  (n=4031) | | |
| --- | --- | --- | --- | --- | --- | --- |
|  | Non-adjusted | Adjust I | Adjust II | Non-adjusted | Adjust I | Adjust II |
|  | OR 95%CI *P*-value | OR 95%CI *P*-value | OR 95%CI *P*-value | OR 95%CI *P*-value | OR 95%CI *P*-value | OR 95%CI *P*-value |
| ionized calcium | 0.67 (0.56, 0.79) <0.0001 | 0.63 (0.53, 0.76) <0.0001 | 0.78 (0.62, 0.98) 0.0319 | 0.67(0.56-0.80) <0.0001 | 0.64(0.53-0.76) <0.0001 | 0.76(0.64-0.92) 0.0036 |
| ionized calcium group |  |  |  |  |  |  |
| <4.4 | 1.96 (1.62, 2.37) <0.0001 | 2.11 (1.74, 2.56) <0.0001 | 1.68 (1.32, 2.15) <0.0001 | 1.96 (1.62, 2.37) <0.0001 | 2.11 (1.74, 2.56) <0.0001 | 1.71 (1.38, 2.11) <0.0001 |
| >=4.4, <5.2 | Reference | Reference | Reference | Reference | Reference | Reference |
| >=5.2 | 1.72 (1.12, 2.62) 0.0128 | 1.82 (1.18, 2.79) 0.0063 | 1.92 (1.15, 3.21) 0.0122 | 1.72 (1.12, 2.62) 0.0128 | 1.82 (1.18, 2.79) 0.0063 | 1.67 (1.06, 2.62) 0.0267 |
| *P* for trend | <0.0001 | <0.0001 | <0.0001 | <0.0001 | <0.0001 | <0.0001 |

 Non-adjusted model adjust for: None
Adjust I model adjust for: gender, age, BMI.
Adjust II model adjust for: gender, age, BMI, ethnicity, temperature, respiratory rate, heart rate, MAP, PaO_2_, PaCO_2_, WBC, hemoglobin, total bilirubin, albumin, glucose, BUN, creatinine, sodium, potassium, chloride, dialysis, vasopressor, calcium gluconate/calcium chloride, septic shock, COPD, diabetes, hypertension, CHF, MI, CKD, cirrhosis, cancer, PE, stroke, site of infection, APACHE Ⅳ score and SOFA score.

Supplemental Table 3. The addressing of nonlinear association between ionized calcium and 28-day mortality

| Models | With missing data  (n=12070) | | Dealing with missing data  (n=13730) | |
| --- | --- | --- | --- | --- |
|  | OR (95%CI) | *P* value | OR (95%CI) | *P* value |
| Model I | | | | |
| One line effect | 0.78 (0.62, 0.98) | 0.0319 | 0.86(0.72,1.04) | 0.1200 |
| Model II | | | | |
| Turning point (K1, K2) | 4.25, 4.65 | | 4.3, 4.7 | |
| Serum Calcium Level < K1 | 0.49 (0.29, 0.82) | 0.0066 | 0.63(0.42,0.93) | 0.0218 |
| Serum Calcium Level K1-K2 | 0.35 (0.08, 1.59) | 0.1732 | 0.37(0.12,1.17) | 0.0907 |
| Serum Calcium Level > K2 | 1.69 (1.05, 2.72) | 0.0307 | 1.62(1.14,2.31) | 0.0069 |
| *P* value for LRT test* |  | <0.001 |  | <0.001 |

Data were presented as OR (95% CI) P value; Model I, linear analysis; Model II, non-linear analysis. Adjusted for: gender, age, BMI, ethnicity, temperature, respiratory rate, heart rate, MAP, PaO_2_, PaCO_2_, WBC, hemoglobin, total bilirubin, albumin, glucose, BUN, creatinine, sodium, potassium, chloride, dialysis, vasopressor, calcium gluconate/calcium chloride, septic shock, COPD, diabetes, hypertension, CHF, MI, CKD, cirrhosis, cancer, PE, stroke, site of infection, APACHE Ⅳ score and SOFA score. *CI* confidence interval, OR odds ratio, LRT logarithm likelihood ratio test. **P*<0.05 indicates that model II is significantly different from Model I.
